# Guided Bone Regeneration with Concentrated Growth Factor Enriched Bone Graft Matrix (Sticky Bone) vs. Bone-Shell Technique in Horizontal Ridge Augmentation: A Retrospective Study

**DOI:** 10.3390/jcm10173953

**Published:** 2021-08-31

**Authors:** Horia Mihail Barbu, Stefania Andrada Iancu, Antonio Rapani, Claudio Stacchi

**Affiliations:** 1 Head of Oral Implantology Department, Faculty of Dental Medicine, Titu Maiorescu University, 031593 Bucharest, Romania; horia.barbu@gmail.com; 2 European Centre of Oral Implantology, 011473 Bucharest, Romania; 3 Department of Prosthodontics, Faculty of Dental Medicine, Titu Maiorescu University, 031593 Bucharest, Romania; 4 Titu Maiorescu Doctoral School of Dental Medicine, 040441 Bucharest, Romania; 5 Department of Medical, Surgical and Health Sciences, University of Trieste, 34129 Trieste, Italy; rapani.antonio@gmail.com (A.R.); claudio@stacchi.it (C.S.)

**Keywords:** bone-shell technique, sticky bone, lateral ridge augmentation, particulate bone grafting, guided bone regeneration, autologous bone chips, xenograft

## Abstract

Background: The purpose of this study was to compare clinical results of two different horizontal ridge augmentation techniques: guided bone regeneration with sticky bone (SB) and the bone-shell technique (BS). Methods: Records of patients who underwent horizontal ridge augmentation with SB (test) and BS (control) were screened for inclusion. Pre-operative and 6-month post-operative ridge widths were measured on cone beam computer tomography (CBCT) and compared. Post-operative complications and implant survival rate were recorded. Results: Eighty consecutive patients were included in the present study. Post-operative complications (flap dehiscence, and graft infection) occurred in ten patients, who dropped out from the study (12.5% complication rate). Stepwise multivariate logistic regression analysis showed a significant inverse correlation between the occurrence of post-operative complications and ridge width (*p* = 0.025). Seventy patients (35 test; 35 control) with a total of 127 implants were included in the final analysis. Mean ridge width gain was 3.7 ± 1.2 mm in the test and 3.7 ± 1.1 mm in the control group, with no significant difference between the two groups. No implant failure was recorded, with a mean follow-up of 42.7 ± 16.0 months after functional loading. Conclusions: SB and BS showed comparable clinical outcomes in horizontal ridge augmentation, resulting in sufficient crestal width increase to allow implant placement in an adequate bone envelope.

## 1. Introduction

Implant dentistry was introduced with the purpose of fulfilling the functional demands of the edentulous patient. It was and still is of great benefit to replace mobile prosthesis with fixed implant supported restorations or even to increase the retention of mobile prosthesis with the aid of dental implants. Nowadays, implantology, having evolved with numerous clinical possibilities, can also improve the aesthetic outcomes of the final restoration.

The goal of modern implant dentistry is to restore normal function, overall mastication, aesthetics and phonation, regardless of existing bone resorption in the edentulous ridge [1,2].

Functional and aesthetic improvement begins with the conception of the treatment plan, when the clinician selects among surgical options and decides on the characteristics of the final restoration. Prosthetic-driven treatment plans often recommend bone augmentation procedures in order to create a surgical site where the implant may be inserted in the ideal prosthetic position [2,3]. Implant position is one of the major factors to consider in order to achieve satisfactory aesthetic outcomes in the final restoration [4].

It was demonstrated that about 50% of the alveolar bone width is lost within 12 months after tooth extraction [5,6]. Previous studies analysing the frequency of bone grafting procedures in implant surgery concluded that more than half of the implant sites, both in the lower and upper jaw, required bone augmentation [7,8,9]. Long-term success and proper aesthetics in implant dentistry can be achieved through preservation or reconstruction of an adequate bone volume of the alveolar process [10]. 

Without bone grafting techniques, an atrophic edentulous ridge may allow for tilted, narrow or short implant insertion [1,11,12,13]. Carl Misch classified the implant-supported prosthetic options resulting from the residual bone crest conditions and the number of implants necessary to perform the restoration. The author defined five possibilities of prosthetic rehabilitation, depending on the existing edentulous crest, from implant-supported overdenture to fixed prosthesis, in which only the crown is replaced and emerges from the soft tissue like a natural tooth [14]. Bone augmentation techniques are key to shifting the residual bone crest into a more favourable category from a prosthetic point of view. 

Horizontal and vertical alveolar ridge deficiencies may be treated by means of sinus floor elevation, bone blocks, particulate bone grafting, ridge splitting, inferior alveolar nerve repositioning, distraction osteogenesis, or by a combination of, or variations on, these techniques [15,16,17]. Bone blocks offer the possibility of performing considerable lateral augmentation—a viable option with predictable results. Based on recent studies, implant survival rate varies from 73.8% to 100% for autogenous or homologous bone blocks [18,19]. The harvested bone block may be pure cortical or cortico-cancellous, depending on the depth of the osteotomy and the location of the donor site [15]. The bone-shell technique is a variation of bone block regeneration, where thin cortical plates are fixed by micro-screws to restore alveolar ridge contours, and the gap with the residual bone crest is then filled with particulate autogenous or xenogeneic graft [20,21]. The creation of a stable regenerative environment, with no micromovements and good vascular and cellular supply, is a crucial pre-requisite to achieving predictable ridge contour restoration with excellent dimensional stability over time [20].

Guided bone regeneration (GBR) with particulate graft represents an alternative solution for the reconstruction of atrophic ridges. One of the many challenges the surgeon must face is the stabilisation of blood clot and graft particles in the desired location during the healing period, avoiding micromovements. This difficulty may be overcome by using non-resorbable d-PTFE titanium reinforced membranes, collagen membranes fixed with pins, the tenting screw technique, or autogenous fibrin glue, as used in the “sticky bone” technique [15,22,23,24]. The preparation of autologous concentrated growth factor (CGF)-enriched bone graft matrix (sticky bone), developed over more than five decades (1970–2010), is recognised for its biological and mechanical benefits [25,26,27]. 

The aim of this retrospective study was to compare the clinical outcomes of two surgical approaches for horizontal ridge augmentation, namely, GBR with concentrated growth factor (CGF)-enriched bone graft matrix (sticky bone) and the bone-shell technique.

## 2. Material and Methods

### 2.1. Study Design

The present research was designed as a retrospective cohort study and was reported in accordance with the Strengthening the Reporting of Observational Studies in Epidemiology (STROBE) guidelines [28]. Two different horizontal ridge augmentation techniques were compared: the test group was GBR with CGF-enriched bone graft matrix (sticky bone), while the bone-shell technique was used as the control group. The entire analysis was made using information from medical files and cone beam computed tomographies (CBCTs) of patients treated at the European Center of Implantology, located in Bucharest, Romania. All participants gave their approval to be part of the research and signed an informed consent document. The study followed ethical standards for research involving human subjects, as outlined in the Declaration of Helsinki (2008) and following revisions (Fortaleza 2013). In order to begin the research, an ethical approval (No. UTM03FEB20-MD19) was first obtained from the Institutional Review Board of Titu Maiorescu University (Bucharest, Romania).

### 2.2. Study Population

Medical charts of all patients treated in the study centre with horizontal ridge augmentation procedures using sticky bone (SB) or the bone-shell technique (BS) were screened for potential inclusion in the present study.

Inclusion criteria were the following:

- Age > 18 years without other age or gender restrictions;
- Horizontal ridge augmentation performed with SB or BS;
- Presence of CBCTs taken before surgery and six months after augmentation;
- Native bone crest width <5 mm (measured at 1 mm below the most cranial point of the alveolar crest);
- Implant-supported fixed prosthetic rehabilitation.

Exclusion criteria were the following:

- Untreated or residual periodontal disease;
- Uncontrolled diabetes (HbA1c > 7.5%);
- Antiresorptive therapy;
- Head and/or neck radiotherapy;
- Immunosuppressive therapy;
- Incomplete or unavailable medical and periodontal charts (including radiographs);
- Post-operative complications (e.g., graft infection, flap dehiscence);
- Absence of signed informed consent;

### 2.3. Surgical Protocol

For all procedures, local anaesthesia was administered using articaine with epinephrine 1:100,000 (Ubistesin Forte, 3M ESPE, Seefeld, Germany). A full thickness flap was elevated to ensure access to the recipient and donor site. Multiple cortical perforations of the recipient bed were performed in order to stimulate bleeding from marrow spaces and facilitate osteoprogenitor cell migration and neo-angiogenesis.

A one-stage approach with immediate implant insertion coupled with lateral ridge augmentation was performed when residual bone allowed adequate implant primary stability. After implant insertion in the correct prosthetic position, insufficient bone thickness resulted in dehiscence or fenestration on the buccal aspect of the implant. The surgeon chose between SB and BS to re-establish horizontal bone volumetry and restore an adequate ridge contour. When it was not possible to achieve primary stability, a two-stage approach was selected, and only the horizontal bone augmentation procedure (SB or BS) was performed.

**Figure 1:**
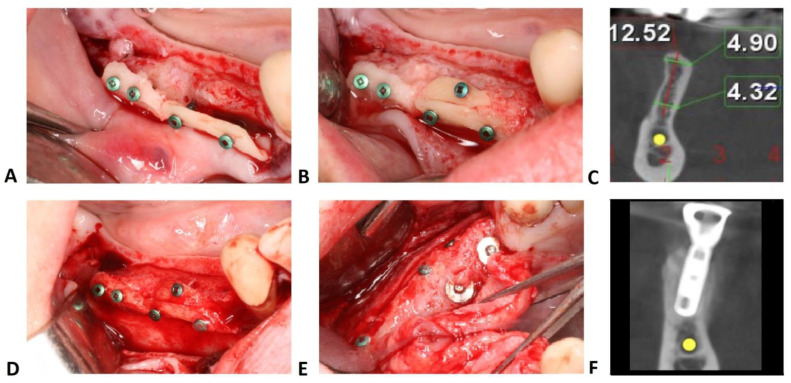
Bone-shell technique. (**A**) Rigid fixation of the bone shells with osteosynthesis screws. (**B**) The gap between bone shell and buccal cortical plate is filled up with autologous bone chips. (**C**) Baseline measurement at 1 mm below the most cranial point of the alveolar crest. (**D**) Second stage surgery after 6 months showing minimal resorption of the autogenous bone particles in the mesial part of the surgical site. (**E**) Implants uncovering after additional 3 months. (**F**) CBCT cross-section after implant osseointegration.

The bone-shell technique was performed by harvesting a cortico-cancellous block from the mandibular retromolar area (Figure 1). Bone cutting was performed using OT7s micro-saw tips from an ultrasonic bone surgery device (Piezosurgery, Mectron, Carasco, Italy) or special diamond disks (Frios MicroSaw, Dentsply Sirona, Charlotte, NC, USA). After bone block outlining, bone was removed by applying slight pressure with chisels on the osteotomy slot. Bone blocks were longitudinally split into two thinner blocks: for large defects, both bone blocks were used and fixed, whilst for smaller defects, only one block was used with the other block fixed back into the donor site with 1 or 2 osteosynthesis screws. Sharp edges were smoothened with a round diamond bur in order to prevent future soft tissue perforations.

After adaptation, the harvested bone segment was placed into the desired position and perforated with a 0.8 mm drill, together with the cortical buccal bone plate. Usually, two osteosynthesis screws with a 1.0 mm diameter and 7–9 mm length were necessary for a rigid fixation of the bone block at a 3–4 mm distance from the buccal plate. The empty space between the bone block and the buccal wall of the edentulous ridge was filled with autologous bone chips harvested from the same area using specific drills (Auto Chips Maker, Neo-Biotech, Seoul, South Korea).

In maxilla, bone blocks were harvested using OT7s micro-saw tips from the lateral wall of the sinus when a sinus augmentation was planned, or from the zygomatic buttress when only lateral bone augmentation was performed. The bone block was perforated and fixed in the same manner as in the mandible. However, in maxilla, longer screws were used (at least 9 mm) to ensure a rigid fixation of the block. 

Sticky bone graft is a mixture of centrifuged autogenous plasma and graft particles (autogenous bone or a composite graft with 80% autogenous bone and 20% anorganic bovine bone). In the lower jaw, autogenous bone chips were harvested from the mandible body just below the receiving site with specific drills (Auto Chips Maker, Neo-Biotech, Seoul, South Korea). In maxilla, autologous bone chips were harvested from the tuberosity, using a bone rongeur forceps (Devemed, Tuttlingen, Germany), due to reduced bone density. When the second and third molar were present, bone chips were harvested from the lateral wall of the sinus by using a bone scraper, or a narrow-diameter ACM drill was used in anterior part of the maxilla, above or between the roots of the dental elements.

Graft particles (autogenous and bovine) were permanently hydrated in saline solution. After harvesting autogenous bone chips, patient venous blood (10–20 mL) was drawn in non-coated vacutainers and then centrifuged for 12 min at 2700 rpm to obtain autologous fibrin glue (AFG). Bone particles were then placed in specific rectangular metal storage boxes, and AFG, after being extracted with a syringe, was added to the bone chips. After agglutination (5–10 min), the two components were firmly united in a compound with gelatinous consistency.

**Figure 2:**
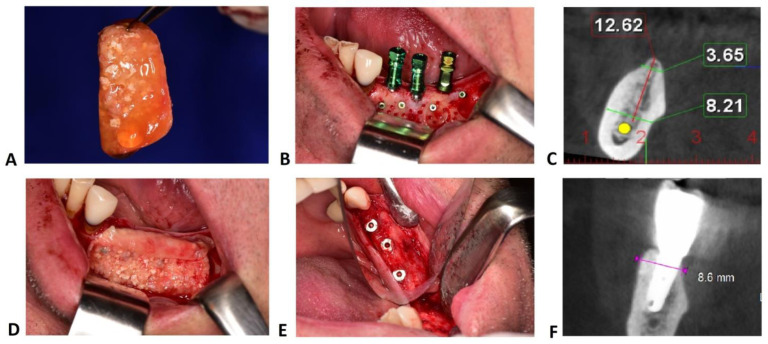
Sticky bone technique. (**A**) Sticky bone, a solid aggregate composed by particulate xenograft mixed with autograft, immersed in fibrin gel concentrated with growth factors, moulded in the desired shape. (**B**) Recipient bed prepared with retentive osteosynthesis screws and implants inserted in the correct prosthetic position. (**C**) Preoperative measurements on CBCT sections of the area of interest. (**D**) Bone graft material hanged on the osteosynthesis screws. (**E**) Regeneration area uncovering after 6 months. (**F**) Post-operative CBCT of the augmented ridge with sticky bone.

While the sticky bone set, osteosynthesis screws were fixed perpendicularly to the buccal plate, leaving them about 3–4 mm distance apart. On these screws, the agglutinated bone was gently pressed and moulded into the bone defect (Figure 2). Two or three osteosynthesis screws were usually necessary for safe fixation of sticky bone and for the adequate space-making effect during the entire healing period.

Different membranes have been used to cover the augmentation sites: autologous platelet-rich fibrin (PRF) membranes were selected where the defect was grafted only by autogenous bone, whilst low-resorption pericardium membranes (CopiOs, Zimmer Biomet, Warsaw, IN, USA) were chosen if xenograft granules had also been used.

Flaps were then mobilised to ensure a passive closure over the regeneration area by longitudinal periosteal incisions and, in the mandible, by using previously described flap releasing techniques [29,30,31]. Horizontal mattress sutures coupled with single stitches or Sentineri sutures were used to attain primary closure of the flaps by using synthetic monofilaments [32].

As part of the postoperative protocol, specific medication was prescribed. All patients were given non-steroidal anti-inflammatory drugs (dexketoprofen 25 mg), usually taken only if needed on the day of the surgery, antibiotics (amoxicillin and clavulanic acid 1 g every 12 h for seven days) and steroidal anti-inflammatory drugs (dexamethasone sodium phosphate 8 mg 1 day before the surgery, 8 mg on the day of surgery and 4 mg the next day). Patients were recalled for control at 3, 7, 10 days (suture removal) and 3 weeks postop. Postoperative control CBCT was performed 6 months after surgery.

### 2.4. Radiographic Measurements

All CBCT measurements were taken by a single calibrated examiner (S.A.I.) on a 26-inch colour diagnostic display. Ridge width in the site of interest was measured at 1 mm below the most cranial point of the alveolar crest. Each measurement was repeated three times at three different time points, as proposed by Gomez-Roman and Launer [33]. Examiner calibration was performed in two calibration sessions held prior to the beginning of the study on a sample of 10 anonymised CBCTs. The second session took place two weeks after the first one, and intra-class correlation coefficient (ICC) was used to assess intra-examiner reliability [34].

### 2.5. Statistical Analysis

Data were analysed using statistical software (IBM SPSS Statistics for Windows, Version 25.0., IBM Corp, Armonk, NY, USA) and patient was considered as the statistical unit. Based upon data published in previous studies, sample size was calculated assuming a difference in horizontal bone gain between the two groups of 1.0 ± 1.3 mm. A minimum sample of 28 subjects for each experimental group was needed to detect significant differences (confidence level 5% with statistical power of 80%). Data for descriptive statistics were expressed as mean ± standard deviation. Dataset normality was evaluated by using Shapiro-Wilk test and homogeneity of variance was analysed by means of Levene test. Two-tailed *t*-test was performed to analyse parametric data, whilst Mann Whitney U test and Chi-Square test were used to assess comparisons of non-parametric data. Univariate logistic regression analysis was first performed to select factors associated with the presence of post-operative complications. Predictor variables resulting significant at the univariate analysis were then inserted in a stepwise multivariate logistic regression model, setting a *p*-value of 0.157 in the stepwise backward model as suggested by Heinze and Dunkler (2017) [35].

## 3. Results

Eighty consecutive surgeries (40 test; 40 control), which were performed between 2012 and 2019 by the same experienced surgeon (H.B.), were included in the present study. Test group patients (28 female–12 male) and control group patients (29 female–11 male) showed no significant differences in terms of gender distribution (chi square *p* = 0.8). Mean age of the entire sample was 58.3 ± 13.4 years (test 51.0 ± 11.9 years–control 47.4 ± 9.7 years). Age was normally distributed between groups according to the Shapiro–Wilk test, and then a parametric test was used (a two-tailed *t*-test). No significant difference in age distribution was demonstrated between test and control groups (*p* = 0.14). Thirty-two surgeries were performed in smokers (17 in test and 15 in control group), and a history of periodontitis was present in 25 patients (12 in test and 13 in control group). No significant difference for these two variables was demonstrated between test and control groups (smoking status: *p* = 0.65; history of periodontitis *p* = 0.81). Demographic information is summarised in Table 1.

Postoperative complications were recorded in ten patients, who dropped out from the study (12.5% complication rate). Three flap dehiscence and two graft infections were recorded in the test group (*n* = 5; two flap dehiscence in the mandible, two infections and one dehiscence in the maxilla). Three flap dehiscence and two graft infections were also recorded in the control group (*n* = 5, all in the mandible). Stepwise multivariate logistic regression analysis showed a significant inverse correlation between the occurrence of postoperative complications and ridge width (OR = 0.249; (95%CI: 0.074–0.837); *p* = 0.025). Complete results of univariate and multivariate analyses are reported in Table 2.

Patients who experienced postoperative complications received a total of 20 implants (11 in test, 9 in control group). Twelve of these implants (60%) were lost or removed (seven in test group (63.6%); five in control group (55.6%)) immediately after the complication onset or within the first 6 months of functional loading. 

Seventy patients (35 test; 35 control) were then included in the final analysis. Twenty-six surgical sites were located in the maxilla (12 test; 14 control) and 44 sites in the mandible (23 test; 21 control), with no significant difference in terms of topographic distribution between the two groups.

Mean crestal width at baseline was not significantly different between the two groups (test: 3.7 ± 0.8 mm; control: 3.0 ± 0.6 mm; two-tailed Mann–Whitney U test *p* = 0.09). After six months of healing, mean crestal width was 7.4 ± 1.1 mm and 6.7 ± 1.2 mm in test and control groups, respectively (two-tailed Mann–Whitney U test *p* = 0.05). Mean gain in terms of horizontal ridge dimension was 3.7 ± 1.2 mm (test; range 0.9–6.2 mm) and 3.7 ± 1.1 mm (control; range 1.5–6.3 mm), with no significant difference between groups (two-tailed Mann–Whitney U test *p* = 0.69). ICC score for radiographic measurements (>0.94) resulted in an excellent intra-examiner repeatability: mean difference in crestal width measurement was 0.11 mm. Radiographic measurements are summarised in Table 3.

A total of 127 implants were inserted in regenerated areas (70 implants in test group; 57 implants in control group) and were followed after functional loading for a mean period of 42.7 ± 16.0 months (range 12 to 84 months). No implants were lost during the entire observation period both in test and control groups (mean follow-up 43.3 ± 15.7 months and 41.9 ± 16.4 months, respectively).

## 4. Discussion

Careful evaluation of available bone volume in future implant sites is a crucial step during implant treatment planning. In particular, horizontal ridge dimension should be regarded as one of the main parameters influencing peri-implant marginal bone stability. Previous studies demonstrated that, after implant insertion, buccal bone thickness ≥1.8–2.0 mm is necessary to prevent marginal bone resorption and subsequent soft tissue recession [36,37,38]. Unfortunately, both maxillary and mandibular edentulous sites very often present insufficient buccolingual width to allow standard implant placement in an adequate horizontal bone envelope [8,9,39]. Hence, horizontal ridge augmentation techniques are often required as an essential part of the treatment plan to ensure positive medium- and long-term clinical outcomes of implant-supported rehabilitations. Limitations, benefits and disadvantages of the existing bone augmentation techniques should always be considered in order to understand and follow the best clinical indications for each individual case. Various surgical approaches are available to increase horizontal ridge dimension (e.g., GBR with resorbable or not resorbable membranes associated with bone substitutes; ridge expansion or split crest; and autogenous, homologous and xenogeneic bone blocks) [40]. Autologous bone block techniques may still be regarded as the gold standard for the osteoconductive, osteoinductive and osteogenetic properties of the graft and for high mechanical stability of the regeneration area—fundamental pre-requisites for new bone formation [41]. The bone-shell technique represents a predictable option for horizontal ridge augmentation, with a low incidence of complications and high implant survival rate [20,42]. However, this surgical approach implies higher morbidity related to the presence of a donor site, together with possible additional complications, such as damages to the inferior alveolar nerve during block harvesting. In the attempt to overcome these limitations, CGF-enriched bone graft matrix (sticky bone) has been proposed as an enhancement of standard GBR technique [22]. Sticky bone has been defined as a frame, composed of bone particles and concentrated growth factors [43]. The mechanical properties of this aggregate facilitate its placement and stabilisation in the bone defect, where it can be moulded into the desired shape [26,43].

The present study compared GBR with CGF-enriched bone graft matrix (sticky bone) and the bone-shell technique. After healing, both techniques assured a surgical site allowing implant osseointegration in an adequate horizontal bone envelope (mean ridge width after healing 7.4 ± 1.1 mm and 6.7 ± 1.2 mm for test and control groups, respectively). No significant difference in terms of mean horizontal bone gain after six months of healing was demonstrated between SB (3.7 ± 1.2 mm) and BS (3.7 ± 1.1 mm). This outcome is in accordance with recent randomised clinical trials demonstrating no significant differences in mean horizontal bone gain between standard guided bone regeneration and bone blocks, even given an allogeneic origin [40,44]. Moreover, data from the present study are consistent with a recent meta-analysis including 35 articles, which reported mean bone gain of 2.6 ± 0.2 mm after guided bone regeneration and 4.0 ± 0.5 mm when using bone blocks [45]. Implants inserted in sites augmented with both techniques demonstrated excellent medium-term survival rate (100%), with a mean follow-up of 42.7 ± 16.0 months after functional loading. This outcome is in perfect accordance with the clinical trials by Amorfini et al. (2014) and Mendoza-Azpur et al. (2019) and with the retrospective studies by Barbu et al. (2016) and by Korsch and Peichl (2021) [44,45,46,47,48].

Incidence of postoperative complications recorded in the present study (12.5% both for test and control groups) is comparable to the weighted complication rate reported in recent systematic reviews on GBR procedures (16.1–16.8%) [49,50]. Moreover, the present findings suggest that implant failure rate in sites showing flap dehiscence and/or graft infection is extremely high (60%). These outcomes indicate high percentages of adverse events and failures for these surgical techniques, even when performed by experienced operators with an adequate learning curve. In daily clinical practice, it should be carefully considered that even higher failure rates can be reasonably expected when these techniques are performed by unexperienced clinicians who only occasionally face this type of surgery. In the present study, multivariate analysis demonstrated significant inverse correlation between the occurrence of postoperative complications and ridge width. This finding could be explained by the fact that narrower ridges require greater horizontal regeneration with the use of greater amounts of graft, which increase the difficulty to obtain flap primary closure over the augmentation area, even when appropriate surgical techniques for flap advancement and passivation have been performed [29,30,31]. No significant effect of other possible influencing factors (smoking, history of periodontitis, surgical technique and jaw area) was demonstrated in the present sample.

The main limitations of the present study are related to the retrospective design and the characteristics of the examined sample: as the records analysed were not specifically collected for the study, some available data may be of low quality and/or potential confounding factors may not have been properly controlled for. Moreover, only quantitative analyses could be performed, as no histological evaluation was conducted. Hence, results from this study should be interpreted with caution, and their generalisability considered limited due to the high operator influence on the outcomes of both test and control surgical techniques.

## 5. Conclusions

Within the limitations of this study, we can conclude that SB and BS showed comparable clinical outcomes in horizontal ridge augmentation, resulting in sufficient crestal width increase to allow implant placement in an adequate bone envelope. Further prospective clinical and histological studies are needed to confirm the present findings and to provide an in-depth assessment of the quality of the regenerated tissue.

## Figures and Tables

**Table 1:** Demographic characteristics of the included patients.

	Test	Control	Significance
**Age**			
Years	51.0 ± 11.9	47.4 ± 9.7	NS
**Gender**			
Male	12	11	NS
Female	28	29	
**Smoking Status**			
Smoker	17	15	NS
Non Smoker	23	25	
**History of Periodontitis**			
Yes	12	13	NS
No	28	27	

Age is expressed as mean ± standard deviation. NS: not significant.

**Table 2:** Univariate and multivariate stepwise logistic regression analysis for the outcome “Post-operative Complications”.

Number of Cases = 80	Univariate Analysis			Multivariate Analysis		
**Post-op Complications**	OR	[95% CI]	*p*-value	OR	[95% CI]	*p*-value
**Age**	0.980	[0.921–1.043]	0.530			
**History of Periodontitis**	0.935	[0.221–3.961]	0.927			
**Gender**	0.214	[0.054–0.848]	0.028 *	0.504	[0.047–0.887]	0.074
**Smoking**	1.593	[0.421–6.020]	0.493			
**Jaw Area** **(maxilla/mandible)**	1.296	[0.308–5.461]	0.724			
**Ridge Width**	0.264	[0.087–0.802]	0.019 *	0.249	[0.074–0.837]	0.025 *
**Surgical Technique** **(SB/BS)**	1.000	[0.266–3.763]	1.000			

OR = Odds Ratio; CI = Confidence Interval; SB = sticky bone; BS = bone shell; * = statistically significant.

**Table 3:** Bone width measurements performed on CBCT cross-sections at 1 mm below the most cranial point of the alveolar crest. Except for maximum width gain, values are expressed as mean ± standard deviation.

	Sticky Bone	Bone-Shell Technique	Significance
**Preoperative Ridge Width (mm)**	3.7 ± 0.8	3.0 ± 0.6	NS
**Post-operative Ridge Width (mm)**	7.4 ± 1.1	6.7 ± 1.2	NS
**Ridge Width Gain (mm)**	3.7 ± 1.2	3.7 ± 1.1	NS
**Maximum Width Gain (mm)**	6.2	6.3	-

## Data Availability

The data presented in this study are available on request from the corresponding authors. Publicly data sharing is not applicable to this article due to privacy policy.
